# Outcomes associated with allogeneic hematopoietic stem cell transplantation for relapsed and refractory Hodgkin lymphoma in the era of novel agents

**DOI:** 10.1002/cam4.5631

**Published:** 2023-01-18

**Authors:** Muhammad Salman Faisal, Walter Hanel, Timothy Voorhees, Rui Li, Ying Huang, Abdullah Khan, David Bond, Yazeed Sawalha, John Reneau, Lapo Alinari, Robert Baiocchi, Beth Christian, Kami Maddocks, Yvonne Efebera, Sam Penza, Ayman Saad, Jonathan Brammer, Marcos DeLima, Samantha Jaglowski, Narendranath Epperla

**Affiliations:** ^1^ Division of Hematology The James Cancer Hospital and Solove Research Institute The Ohio State University Columbus Ohio USA; ^2^ Division of Hematology and Medical Oncology Roswell Park Cancer Institute Buffalo New York USA; ^3^ Division of Hematology and Oncology OhioHealth Bing Cancer Center Columbus Ohio USA

**Keywords:** allogeneic hematopoietic cell transplantation, Allo‐HCT, Hodgkin lymphoma, relapsed/refractory, survival

## Abstract

**Background:**

Relapsed or refractory Hodgkin lymphoma (R/R HL) is a challenging disease with limited treatment options beyond brentuximab vedotin and checkpoint inhibitors. Herein we present the time‐trend analysis of R/R HL patients who received allogeneic hematopoietic cell transplantation (allo‐HCT) at our center from 2001–2017.

**Methods:**

The patients were divided into two distinct treatment cohorts: era1 (2001–2010), and era2 (2011–2017). The primary endpoint was overall survival (OS). Secondary endpoints included progression‐free survival (PFS), non‐relapse mortality (NRM), and cumulative incidence of acute and chronic graft versus host disease (GVHD).

**Results:**

Among the 51 patients included in the study, 29 were in era1, and 22 were in era2. There was decreased use of myeloablative conditioning in era2 (18% vs. 31%) compared to era1 and 95% of patients in era2 previously received brentuximab Vedotin (BV). Haploidentical donors were seen exclusively in era2 (0% vs. 14%) and more patients received alternative donor transplants (7% vs. 32%) in era2. The 4‐year OS (34% vs. 83%, *p* < 0.001) and 4‐year PFS (28% vs. 62%, *p* = 0.001) were significantly inferior in era1 compared to era2. The incidence of 1‐year NRM was lower in era2 compared to era1 (5% vs. 34%, *p* = 0.06). The cumulative incidence of acute GVHD at day 100 was similar in both eras (*p* = 0.50), but the incidence of chronic GVHD at 1 year was higher in era2 compared to era1 (55% vs. 21%, *p* = 0.03).

**Conclusions:**

Despite the advent of novel therapies, allo‐HCT remains an important therapeutic option for patients with R/R HL.

## Graphical abstract

R/R HL after auto‐HCT remains a therapeutic challenge despite newer therapies. Our results show significant incremental improvements in PFS, OS, and NRM over the last two decades at our institution in allo‐HCT recipients.

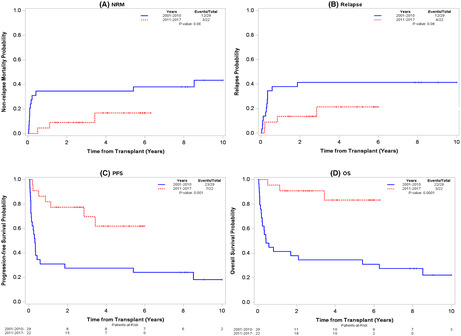

## 1 INTRODUCTION

Hodgkin lymphoma (HL) is a B‐cell lymphoid malignancy accounting for 10% of all lymphomas in the United States with an estimated 8480 new cases and 970 deaths in 2020.^1^ The majority of patients achieve remission with first‐line treatment with combined modality chemotherapy and involved site radiation therapy or chemotherapy alone.^2^ In relapsed or refractory (R/R) HL, salvage with chemotherapy and autologous hematopoietic cell transplantation (auto‐HCT) can be curative for patients with chemosensitive disease.^3^

Treatment options for patients who relapse following auto‐HCT include brentuximab vedotin (BV) and checkpoint inhibitors (CPI) such as pembrolizumab and nivolumab. In a pivotal phase II trial using BV in relapse after auto‐HCT in HL, 41% of patients achieved a complete remission (CR); however, this CR was sustained only in nine patients (9% of the enrolled population) at 5 years who did not receive consolidation with allogeneic HCT (allo‐HCT) or further therapy.^4^ In the KEYNOTE‐87 trial, the overall response rate (ORR) and CR rate were 71% and 28%, respectively in patients treated with pembrolizumab following auto‐HCT relapse with a median duration of response (DOR) of 16.5 months.^5^ However, despite these advances, patients who progress following auto‐HCT normally do not achieve long‐term disease control without the use of allo‐HCT.

Allo‐HCT has been utilized for relapsed/refractory (R/R) HL since the 1980s.^6^ While the initial studies showed good response rates with myeloablative conditioning (MAC) allo‐HCT, the non‐relapse mortality (NRM) was unacceptably high.^7^ With the advent of reduced‐intensity conditioning (RIC) allo‐HCT, the NRM rate dropped substantially without affecting efficacy.^8^, ^9^ In a European Society for Blood and Marrow Transplantation (EBMT) analysis of the patients undergoing allo‐HCT for HL between the years 1997–2001 (*n* = 168, MAC, 79, and RIC, 89), NRM significantly decreased in the RIC group (hazard ratio [HR], 2.85; *p* < 0.001) along with a significant improvement in overall survival (OS) (HR, 2.05; *p* = 0.04) compared to the MAC cohort.^10^ However, in a more recent cohort of patients (2006–2010, *n* = 312, MAC = 63, and RIC = 249), MAC and RIC cohorts showed similar OS, progression‐free survival (PFS), and NRM likely reflective of better patient selection, and/or improved supportive care in recent years.^10^ In the HDR‐ALLO phase II study, 78 out of 92 enrolled patients underwent RIC allo‐HCT with PFS of 47% and OS of 71% at 1 year. The NRM was only 8% at 100 days.^11^, ^12^

While allo‐HCT is a potentially curative option in R/R HL patients, there is a paucity of data looking at the long‐term rate of utilization and outcomes following allo‐HCT, especially in the era of novel agents. Hence, we sought to evaluate the transplant characteristics, clinical outcomes, and utilization of allo‐HCT among patients with HL using data from our transplant registry.

## 2 PATIENTS AND METHODS

### 2.1 Study design and patients

This is a single‐center retrospective study of R/R HL patients receiving allo‐HCT at the Ohio State University. All patients ≥18 years of age receiving an allo‐HCT for R/R HL between 2001 and 2017 were identified from our transplant registry by using data codes for HL. Data on age, conditioning, donor source, graft versus host disease (GVHD), relapse rate, disease status at transplant, prior lines of therapy, and post‐transplant outcomes were extracted using a database query. Missing data from the database query were retrieved through medical record review. Patients were stratified into two eras (era1, 2001–2010; era2, 2011–2017) based on the date of transplant. Disease status prior to transplant was measured by PET scan in era2 while era1 utilized CT and/or PET scan. The year 2011 was chosen as the cut‐off due to the availability of BV starting that year.

Reduced‐intensity conditioning (RIC) regimens included fludarabine and busulfan (Flu/Bu) with a target area under the curve of 4000 uMol/min for 4 days or fludarabine and melphalan (Flu/Mel, 140 mg/m^2^) or fludarabine, cyclophosphamide, and total body irradiation (Flu/Cy/TBI). Matched related donor (MRD) recipients received tacrolimus and methotrexate (FKMTX) while matched unrelated donor (MUD) recipients received FKMTX with pre‐transplant rabbit anti‐thymocyte globulin (ATG) till 2016. All cord blood recipients received tacrolimus and mycophenolate (FKMMF) while haploidentical transplant (haplo‐HCT) recipients received FKMMF with post‐transplant cyclophosphamide (PTCy).

### 2.2 Study endpoints and definitions

The primary endpoint was OS. Secondary endpoints included PFS, NRM, and cumulative incidence of acute and chronic graft versus host disease (GVHD).

Neutrophil recovery was defined as absolute neutrophil count (ANC) ≥500/μl for at least 3 consecutive days after post‐transplantation nadir. Platelet recovery was defined as achieving platelet counts ≥20,000/μl for at least 3 days, unsupported by transfusion. Acute GVHD and chronic GVHD were graded using standard criteria.^13^, ^14^ NRM was defined as death from any cause in the absence of lymphoma progression and calculated from the date of allo‐HCT to the date of death. OS was determined from the date of allo‐HCT to the date of death due to any cause; patients who were alive were censored at the date of the last follow‐up. PFS was calculated from the date of allo‐HCT to the date of disease progression or death, whichever occurred first. Patients who were alive and progression‐free were censored at the date of the last follow‐up.

### 2.3 Statistical analyses

Baseline characteristics were compared across the two eras. Categorical variables are presented as counts and percentages while numeric variables were presented as a median with range. Statistical differences were determined by Chi‐Square or Fisher's exact test for categorical variables and the Wilcoxon Rank‐sum test for numeric variables. The probabilities of OS and PFS were estimated using the Kaplan–Meier method. Log‐rank test was applied to evaluate differences among the three groups. For the calculation of NRM, relapse was viewed as a competing risk. For acute and chronic GVHD, relapse, and death without GVHD were considered as competing risks. Cumulative incidence of NRM, relapse and GVHD were estimated using the cumulative incidence function and compared between groups using the Fine and Gray method. All analysis was performed in SAS software version 9.4.

## 3 RESULTS

### 3.1 Baseline characteristics

Among the 51 patients who met the inclusion criteria, 29 patients were in era1, and 22 patients were in era2 (2011–2017). Table 1 describes the baseline characteristics of the patients stratified by the era of transplant. The median age was similar across the two eras. The median number of prior treatments was four and five in era1 and era2, respectively. Most of the patients had chemosensitive disease (64% in era1 vs. 69% in era2). A larger percentage of patients in era2 received prior auto‐HCT (82%) versus era1 (66%). In era2, seven patients (32%) received alternate donor transplant, with four receiving cord blood transplant and three receiving haplo‐HCT, while in era1, only two patients received cord blood transplant, and none received haplo‐HCT. In era2, 95% had received prior BV, while 14% received prior nivolumab at the time of allo‐HCT. The conditioning regimens in era1 and era2 were mostly RIC (68% vs. 82% in eras 1 and 2, respectively). The most commonly used GVHD prophylactic regimen was calcineurin inhibitor with methotrexate while mycophenolate and cyclophosphamide were the most common alternative regimens. All patients who underwent haplo‐HCT received PTCy for GVHD prophylaxis. The median duration of follow‐up was 9.06 years for era1, and 4.07 years for era2.

**TABLE 1:** Baseline characteristics of patients who received allogeneic stem cell transplant for Hodgkin disease in the years 2001–2017.

Characteristics	2001–2010 (*N* = 29, %)	2011–2017 (*N* = 22, %)	*p*‐value
Age at HCT, median (range) in years	33 (22–62)	33.5 (19–64)	0.66
Gender of recipient			0.64
Female	12 (43)	10 (45)	
Male	16 (57)	12 (55)	
Race of recipient			0.39
African American	2 (7)	0	
Caucasian	26 (93)	21 (95)	
Others	0	1 (5)	
Conditioning regimen^a^			<0.0001
MAC	9 (31)	4 (18)	
RIC	20 (69)	18 (82)	
Donor type			0.03
Related	14 (48)	5 (23)	
Unrelated	13 (45)	10 (45)	
Cord	2 (7)	4 (18)	
Haplo	0	3 (14)	
Stem cell source			<0.0001
BM	2 (7)	1 (5)	
CB	2 (7)	4 (18)	
PB	25 (86)	17 (77)	
Remission status at transplant			0.03
CR	3 (11)	7 (32)	
PR	15 (54)	8 (36)	
Chemoresistant	10 (36)	7 (32)	
Prior brentuximab exposure	0	21 (95)	<0.0001
Prior Nivolumab exposure	0	3 (14)	0.04
KPS at transplant			<0.01
<90	12 (44)	4 (18.18)	
≥90	15 (56)	18 (82)	
Number of patients with prior auto‐HCT	19 (66)	18 (82)	<0.0001
Median lines of therapy, median (range)	4 (2–7)	5 (4–9)	<0.01

Abbreviations: BM, bone marrow; CB, cord blood; CR, complete remission; MAC, myeloablative conditioning; PB, Peripheral blood; PR, partial remission; RIC, reduced intensity conditioning.

^a^ MAC regimens were BuCY or FluBu, RIC were FluBu, FluMel, FluCyTBI and others. Bu, Busulfan; Cy, Cyclophosphamide; Flu, fludarabine; Mel, Melphalan.

### 3.2 Hematopoietic recovery

The median time to neutrophil engraftment was 13.5 (range = 0–27), and 15.5 (range = 6–19) days across eras 1 and 2, respectively (*p* = 0.08). The median time to platelet engraftment was 20 (range = 0–83), and 17 days (range = 0–46) (*p* = 0.21) (Table 2).

**TABLE 2:** Outcomes of allo‐HCT for Hodgkin lymphoma in two eras.

	2001–2010 (*N* = 29, 95% CI)	2011–2017 (*N* = 22, 95% CI)	*p*‐value
Time to ANC engraftment in days, median (range)	13.5 (0–27)	15.5 (6–19)	0.08
Time to platelet engraftment in days, median (range)	20 (0–83)	17 (0–46)	0.21
Incidence of aGVHD			0.51
Day 100 estimate	0.48 (0.29–0.65)	0.59 (0.35–0.77)	
Day 180 estimate	0.52 (0.32–0.68)	0.68 (0.43–0.84)	
Incidence of severe aGVHD			0.15
Day 100 estimate	0.10 (0.03–0.25)	0.00 (0.00–0.00)	
Day 180 estimate	0.17 (0.06–0.33)	0.05 (0.00–0.19)	
Incidence of cGVHD			0.03
1‐year estimate	0.21 (0.08–0.37)	0.55 (0.31–0.73)	
NRM			0.06
1‐year estimate	0.34 (0.18–0.52)	0.05 (0.00–0.19)	
4‐year estimate	0.34 (0.18–0.52)	0.17 (0.04–0.38)	
Incidence of Relapse			0.08
1‐year estimate	0.38 (0.20–0.55)	0.14 (0.03–0.31)	
4‐year estimate	0.41 (0.23, 0.59)	0.21 (0.06–0.43)	
PFS			0.001
1‐year estimate	0.31 (0.16–0.48)	0.82 (0.59–0.93)	
4‐year estimate	0.28 (0.13–0.44)	0.62 (0.34–0.81)	
OS			<0.0001
1‐year estimate	0.41 (0.24–0.58)	0.95 (0.72–0.99)	
4‐year estimate	0.34 (0.18–0.51)	0.83 (0.55–0.95)	

### 3.3 Infectious complications and graft failure

The incidence of bacteremia (15%–19%) and viral reactivation of CMV, EBV, and BK virus (40–50%) within the first 100 days was not statistically different between the two eras. There were no fungal infections in either group. Only two patients had graft failure (3%), with both cases occurring in era1 (Table 3).

**TABLE 3:** Transplant‐related complications after allo‐HCT for Hodgkin lymphoma.

Characteristics	2001–2010 (*N* = 29, %)	2011–2017 (*N* = 22, %)	*p*‐value
Bacteremia in day + 100			1.0
No	17 (80.95)	16 (84.21)	
Yes	4 (19.05)	3 (15.79)	
Viral reactivation in day + 100			0.6
No	11 (52.38)	12 (60.00)	
Yes	10 (47.62)	8 (40.00)	
Fungemia in day + 100			NA
No	21 (100)	20 (100.00)	
Yes	0 (0.00)	0 (0.00)	
Incidence of graft failure			0.49
No	26 (89.66)	22 (100.00)	
Yes	2 (6.90)	0 (0.00)	

### 3.4 Acute and chronic GVHD

**FIGURE 1:**
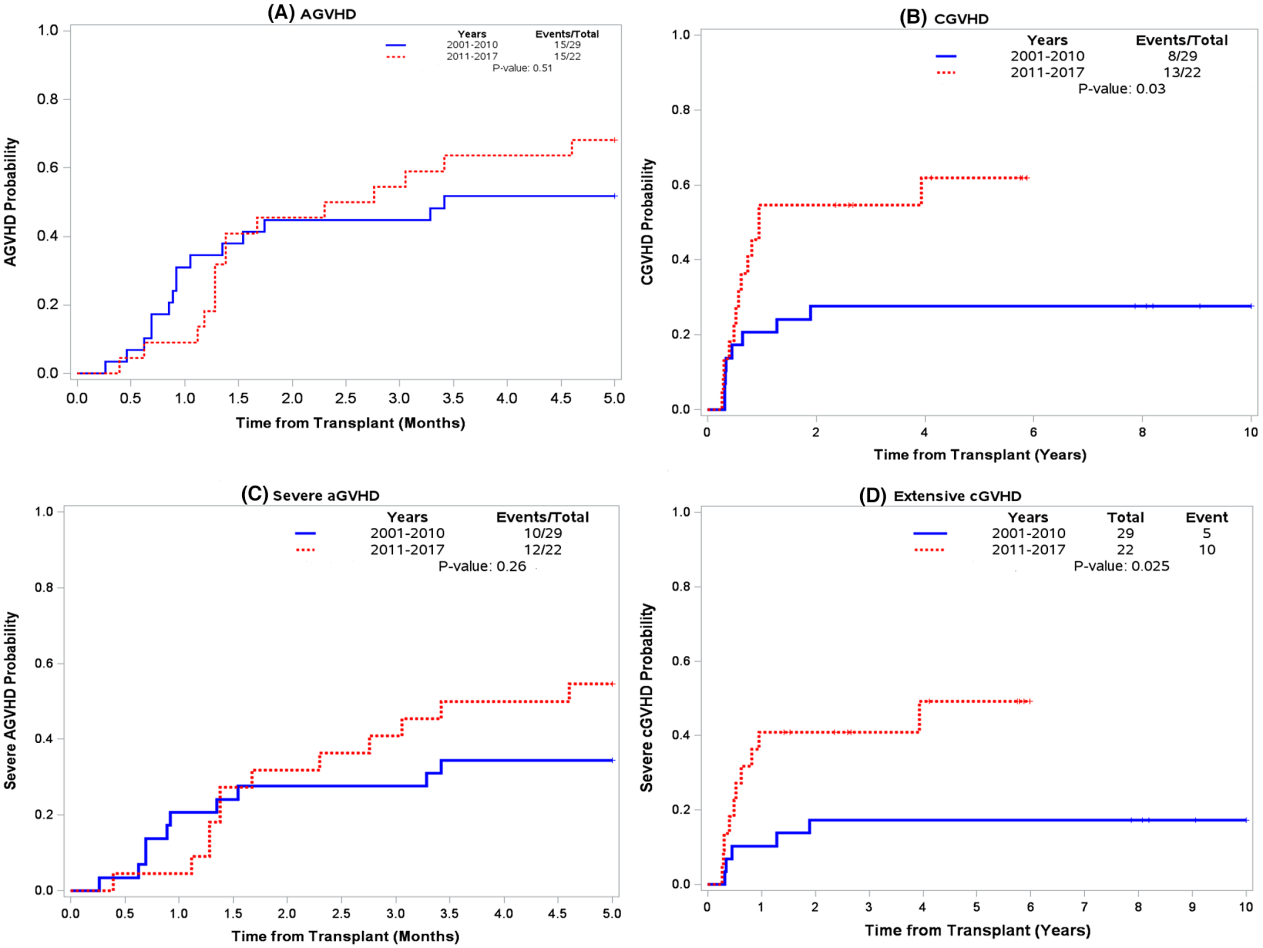
Cumulative incidence of GVHD across two eras (A) Acute GVHD (B) Chronic GVHD (C) Acute grade II‐IV GVHD (D) Extensive chronic GVHD.

The cumulative incidence of acute GVHD at day +180 was 52%, and 68% in eras 1 and 2, respectively (*p* = 0.50, Figure 1A). The incidence of grade II‐IV acute GVHD was 34% versus 54% (*p* = 0.26, Figure 1C). Grade 3–4 acute GVHD was rare in era2 (5%) compared to 17% in era1, though not statistically significant (*p* = 0.15, Table 2). None of the patients with prior exposure to CPI developed grade 3–4 acute GVHD. The estimated incidence of chronic GVHD at 1 year was 21% versus 55% for eras 1 and 2, respectively (*p* = 0.03). (Table 2, Figure 1B **)**. The incidence of extensive chronic GVHD was 17% versus 45% (*p* = 0.025, Figure 1D). The effect of donor type on the incidence of acute GVHD was not determined due to a small number of patients getting haplo‐HCT in different eras.

### 3.5 NRM and relapse

**FIGURE 2:**
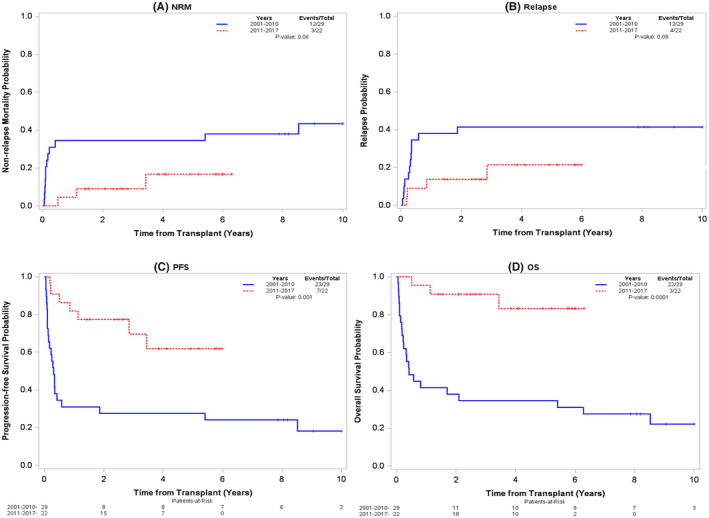
Outcomes across two eras (A) Non‐relapse mortality (B) Relapse (C) Progression‐free survival (D) Overall survival.

The incidence of NRM at 1 year was 34% in era1 versus 5% in era2 (Figure 2A). The four‐year estimated NRM was 34% and 17%, respectively, across the two eras (0.06). The 1‐year cumulative incidence of relapse in era1 was 38% compared to 14% in era2 (*p* = 0.08) (Table 2, Figure 2B).

### 3.6 PFS and OS

The 1‐ and 4‐year PFS rates were 31% and 28% in era1 compared to 82% and 62% in era2 (*p* = 0.001, Table 2, Figure 2C). In era1, the 4‐year PFS was 11% versus 35% in patients who received MAC versus RIC, respectively (RR = 2.19, 95% CI = 0.9–5.3, *p* = 0.07), while in era2, it was NR versus 60% in the recipients of MAC versus RIC (RR = 0.89, 95% CI = 0.11–7.42, *p* = 0.91).

The OS was significantly longer in era2 compared to era1 with estimated 1‐ and 4‐year OS rates of 41% and 34% in era1 versus 95% and 83% in era2 (*p* < 0.0001, Table 2, Figure 2D).

### 3.7 Cause of death

In era1, four patients died due to chronic GVHD, two due to acute GVHD, two due to ARDS, three due to sepsis, and one due to graft failure. Ten patients died due to disease progression. In era2, two patients died due to cGVHD, and one due to acute GVHD. No death due to disease progression was noted in era2 on the date of last follow‐up.

## 4 DISCUSSION

Here we compare our center's transplant experience over the past two decades for patients undergoing allo‐HCT for R/R HL and make several important observations. First, more patients received allo‐HCT as a later line of therapy with addition of BV and CPI before transplant. Second, there was a significant increase in the rates of utilization of RIC regimens in the last decade. Lastly, the incidence of 1‐year NRM decreased over the most recent time period that likely translated to a superior OS in the most recent era.

Allo‐HCT is a time‐tested treatment for R/R HL.^15^, ^16^, ^17^ The long‐term remission in allo‐HCT recipients is predicated on the graft‐versus‐lymphoma effect.^18^, ^19^ The improvement in survival among the patients after the turn of the new millennium is well described in a large meta‐analysis by Rashidi et al.^20^ In this meta‐analysis comparing outcomes of 42 studies with 1850 patients, OS was improved by 10%–20% (*p* < 0.01), and NRM was decreased by 5%–10% (*p* = 0.021) at 1 year in a pooled analysis. We also see an incremental increase in OS and decrease in NRM in era2, compared to era1, with an improvement in OS by 40% and a decline in NRM by 17% at 4 years, which is substantial and represents an improvement in supportive care and better patient selection as shown by more patients in CR at the time of transplant (31% vs. 10%) in era2 versus era1. The disease status at transplant is a well‐known prognostic factor for transplant outcomes.^21^, ^22^, ^23^, ^24^, ^25^, ^26^, ^27^, ^28^ In the meta‐analysis described above, patients with chemo‐sensitive disease had better OS and lower NRM compared to patients with chemo‐resistant disease.^20^ Over the last decade, the emergence of newer therapies has led to an increase in chemosensitivity at the time of allo‐HCT. In our study, 21 of 22 patients in era2 received BV before allo‐HCT and 67% achieved a PR or better response at transplant in line with the previous studies.^29^ The controversy about the use of BV as bridge to transplant versus continuation till disease progression remains unanswered. In the pivotal trial using BV in patients who relapsed after autologous transplant in HL, though 75% of patients had responded to BV, the median PFS was only 5.6 months in all patients and 20.5 months in patients who achieved a CR.^30^ In our series, 11/21 patients were on BV with responsive disease at the time of allo‐HSCT, while other patients needed bridging chemotherapy, of which 6/21 patients had refractory disease. More clinical studies are needed to see the outcomes of BV responsive disease at the time of allo‐HCT.

Most patients in both eras received RIC conditioning, which is consistent with paradigm shift to RIC regimens nationally for patients with lymphoma undergoing allo‐HCT.^11^, ^12^, ^19^, ^31^, ^32^, ^33^, ^34^, ^35^, ^36^ The American Society of Transplant and Cellular Therapy (ASTCT) recommends RIC as the preferred conditioning for allo HCT in HL.^37^ The utility of haplo‐HCT has increased in the last decade with PTCy (Haplo/PTCy) for GVHD prophylaxis.^38^, ^39^ The PTCy leads to milder GVHD by abrogating the CPI induced immune activation^40^ and promoting the vigorous recovery of regulatory T‐cells leading to immune tolerance.^41^ In a recently published “real‐world” analysis of cHL patients who underwent allo‐HCT after CPI treatments, those who received Haplo/PTCy had a lower cumulative incidence of relapse (2‐year CIR = 7%) and excellent OS (2‐year OS = 85%).^42^ We had only three patients in era2 who received haplo‐HCT, compared to none in era1, a number which we expect will increase in the future. The use of alternative donor transplants, especially of haplo‐HCT increased in era2. This is in accord with the increased utility of haplo‐HCT as the preferred method for alternative donor allo‐HCT.^43^ In a report from lymphoma working party of European bone marrow transplant, Eurocord and center of international bone marrow transplant research (CIBMTR), the survival for haplo‐HCT recipients was better than the cord blood transplant recipients (HR 1.55, 4‐year OS and PFS of 58% and 46% vs. 49% and 38%, respectively).^44^

With the arrival of newer therapies such as BV, CPI, and CD30 directed chimeric antigen receptor T‐cell therapy (CD30.CAR‐T), the sequencing of therapies in R/R HL is under constant evolution, and therefore, appropriate timing of allo‐HCT is not well established. In a phase I/II study, CD30.CAR‐T cell therapy was found to be highly effective with an objective response rate of 72%; however, the 1‐year PFS was only 36%.^45^ While CD30.CAR‐T cell therapy is promising, and currently, under investigation (NCT04268706), it is not yet available for most R/R HL patients. Allo‐HCT still is the only curative option in the R/R HL patients who relapse after an autologous stem cell transplant and holds the value of an important therapeutic modality in the management of these patients.

The efficacy of CPI has raised questions on the utility of allo‐HCT in HL. Although CPI provides high ORR, they have a limited duration of response. For instance, in checkmate 205, the median PFS for patients who received nivolumab was 14.7 months, and duration of response was 16.6 months.^46^ Similarly, in KEYNOTE 087 pembrolizumab showed a median duration of response of 16.5 months.^5^ Adding to the complexity of timing in the use of allo‐HCT in R/R HL is the data suggesting that CPI are associated with increased toxicity when used either before or after allo‐HCT. The use of CPI immediately prior to allo‐HCT has been linked to an increased risk of acute GVHD.^47^ The use of CPI after allo‐HCT has been shown to increase the risk of both acute and chronic GVHD.^48^, ^49^ In our study, we did not observe any grade 3–4 acute GVHD in those who received prior CPI therapy, but we had only three patients who received nivolumab prior to transplant, therefore, this data needs to be interpreted with caution given the relatively small numbers of patients included in our analysis. Despite the improvement in NRM over the past two decades, it's not negligible, which brings the question of whether CPIs should be used as a bridge or continue until progression. Further investigation into the effects of CPI before and after allo‐HCT is warranted.

Our study is limited by the retrospective study design where the choice of therapy was at the discretion of the treating physician. Additionally, the small sample size precludes our ability to perform multivariable analysis. Lastly, the small number of patients receiving CPI peritransplant makes it difficult to deduce any conclusions on its impact on GVHD. Notwithstanding these limitations, we noted a trend of improved outcomes associated with allo‐HCT in R/R HL with a 4‐year PFS and OS of 62% and 83% in era2, despite multiple prior lines of therapy.

## 5 CONCLUSION

R/R HL after auto‐HCT remains a therapeutic challenge despite the introduction of newer therapies. Our results show significant incremental improvements in PFS, OS, and NRM over the last two decades at our institution in allo‐HCT recipients. Key factors likely contributing to the improved outcomes include improvement in NRM owing to better supportive care, RIC conditioning regimen, and Haplo/PTCy stem cell grafts, and a higher number of patients achieving chemosensitivity at the time of allo‐HCT due to the availability of more effective therapies in the relapsed setting. This data reaffirms that allo‐HCT should maintain a place in treatment algorithms even in the era of novel agents and cellular therapies.

## AUTHOR CONTRIBUTIONS

**Muhammad Salman Faisal:** Data curation (equal); writing – original draft (lead). **Walter Hanel:** Writing – review and editing (equal). **Timothy Joseph Voorhees:** Writing – review and editing (equal). **Rui Li:** Formal analysis (equal). **Ying Huang:** Formal analysis (equal). **Abdullah Khan:** Writing – review and editing (equal). **David Alan Bond:** Writing – review and editing (equal). **Yazeed Sawalha:** Writing – review and editing (equal). **John Reneau:** Writing – review and editing (equal). **Lapo Alinari:** Writing – review and editing (equal). **Robert Biaocchi:** Writing – review and editing (equal). **Beth A Christian:** Writing – review and editing (equal). **Kami Maddocks:** Writing – review and editing (equal). **Yvonne Adeduni Efebera:** Writing – review and editing (equal). **Sam Penza:** Writing – review and editing (equal). **Ayman Saad:** Writing – review and editing (equal). **Jonathan Brammer:** Writing – review and editing (equal). **Marcos De Lima:** Writing – review and editing (equal). **Samantha M Jaglowski:** Writing – review and editing (equal). **Narendranath Epperla:** Conceptualization (lead); investigation (lead); methodology (lead); writing – original draft (equal); writing – review and editing (lead).

## CONFLICT OF INTEREST

MSF, WH, TV, RL, YH, AK, YS, JR, LA, RB, KM, SP, JB, MD have no relevant COI. DB received consulting/ honoraria from SeaGen and Kite/Gilead. BC received research funding from Celgene, Genentech, Acerta, Triphase, MorphoSys, Merck, SeaGen, Millenium BMS; on advisory Board for Seattle Genetics, Verastem, AstraZeneca, Genentech, MorphoSys. YE is on speakers bureau for Takeda, Oncopeptide, Janssen, Alnylum; received honorarium from Takeda, Oncopeptide, Janssen, GSK, Alnylum, Sanofi; on advisory Board for Takeda, Oncopeptide, Janssen, GSK, Alnylum, Sanofi; received research support from BMS/Celgene. AS is a consultant for Magenta Therapeutics, Incyte Pharmaceuticals, CareDx; received research funding from Amgen, Kadmon, and OrcaBio. SJ received research funding from Kite, Novartis, and Caribou; on advisory board for Kite, Novartis, Juno/BMS, CRISPR, and Takeda. NE receives research funding from Beigene; Speakers Bureau: Incyte; Advisory board/Consultancy/Honorarium: TG Therapeutics, Beigene, SeaGen, Novartis.

## HUMAN STUDY AND SUBJECT STATEMENT

The study was approved by the Institutional review board (IRB) of Ohio State University (OSU). The study conforms to the Declaration of Helsinki.

## CONSENT STATEMENT

A waiver of consent was obtained from IRB at OSU. The waiver was obtained because of the retrospective nature of the study with minimal risk to the subjects and that many of the patients either passed away or had lost to follow‐up since their treatment.

## Data Availability

De‐identified data will be made available on request to the corresponding author.
